# Association between combinations of preoperative comorbidities and postoperative delirium in older patients: a matched cohort study

**DOI:** 10.1186/s12871-025-03110-1

**Published:** 2025-05-15

**Authors:** Ao Li, Yuxiang Song, Wenzhu Shi, Weidong Mi, Jingsheng Lou, Jing Liu

**Affiliations:** 1 Department of Anesthesiology, The First Medical Center of Chinese PLA General Hospital, Beijing, China; 2 National Clinical Research Center for Geriatric Diseases, Beijing, China

**Keywords:** Preoperative comorbidities, Postoperative delirium, Propensity-score matching, Older patients

## Abstract

**Background:**

The current study aimed to investigate which one or certain combinations of preoperative comorbidities were associated with higher risk of postoperative delirium.

**Methods:**

This propensity-score-matched cohort study analyzed a retrospective dataset of elderly patients undergoing surgery at the First Medical Center of the Chinese PLA General Hospital from January 2014 to April 2019. Univariate risk factors were selected by logistic regression, and then the combinations of these univariate factors were compared.

**Results:**

We identified 1034 older patients developed postoperative delirium (POD) within seven days after surgery, and 3102 patients without POD were matched by propensity score matching analysis at a ratio of 1:3 for those with POD. Eight preoperative comorbidities, including hypertension, diabetes, atrial fibrillation, cerebrovascular disease, Parkinson’s disease, epilepsy, depression, and chronic obstructive pulmonary disease (COPD) were more common in patients diagnosed with POD after surgery than those without POD. Patients with POD were more likely to concurrently suffer from the combinations of hypertension and cerebrovascular disease, or hypertension and COPD.

**Conclusions:**

Several preoperative comorbidities are associated with a higher risk of POD in older surgical patient. Those suffering from combinations of preoperative comorbidities were more likely to develop POD, and hypertension plays a central role in these combinations.

## Background

Delirium is one of the most common postoperative complications in older patients, which could be attributed to the vulnerability of the aging brain to surgical and/or anesthetic stress [1, 2]. Preoperative identification of susceptible patients to postoperative delirium (POD) would provide an opportunity for early interference in predisposing risk factors and effective decision-making during perioperative management [3]. Although higher American Society of Anesthesiologists (ASA) physical status and Charlson Comorbidity Index (CCI) were identified as a preoperative risk factor for POD, much less is known about the relationship between specific preoperative comorbidities and the incidence of POD in elderly patients [4, 5].

As a result of an aging population, the preoperative health status of older patients has commonly been diagnosed as multiple comorbidities rather than single diseases [6]. The prevalence of various comorbidities ranged from 3.5 to 100% for older adults worldwide [7]. Assessment of comorbidities for older surgical patients and early prediction of the risk of POD for those with multiple comorbidities is essential to optimize postoperative outcomes and lower healthcare costs [8]. ASA physical status provides a simple tool to predict the preoperative conditions of surgical patients, and the CCI score included a more comprehensive evaluation of comorbidities for physical status assessment [9]. Higher ASA physical status and CCI scores, indicating more preoperative comorbidities, indeed predict a higher risk for POD [10]. However, since these two assessments were designed for an overall health status, neither could distinguish the specific one or more comorbidities associated with the development of POD.

Considering this, the current single-center retrospective cohort study included surgical patients developing POD or not, and matched the perioperative variables related to POD by propensity-score matching to investigate which one or combinations of preoperative comorbidities were associated with the POD. The newly identified preoperative comorbidities and their combinations would benefit the risk assessment of older surgical patients and provide a new perspective for individualized perioperative management for those at higher risk of POD.

## Methods

In this propensity-score-matched cohort study, we analyzed a retrospective dataset of elderly patients undergoing surgery at the First Medical Center of the Chinese PLA General Hospital from January 2014 to April 2019. All methods in the current study were conducted in accordance with the Declaration of Helsinki, and all experimental protocols were approved by the Ethics Committee Board of the First Medical Center of Chinese PLA General Hospital (number: S2019-311-03). Due to the retrospective nature of the study, the institutional review board of the First Medical Center of Chinese PLA General Hospital waived the need of obtaining informed consent.

### Data acquisition

The dataset was established by retrieving perioperative medical records with hospital information system. The inclusion criteria were as follows: (1) age of patients ≥ 65 years and (2) patients undergoing surgery with general anesthesia. The exclusion criteria were as follows: (1) patients undergoing neurosurgery or cardiac surgery, (2) patients undergoing digestive endoscopy, and (3) patients with > 50% of data missing. Preoperative and intraoperative variables relevant to POD were then included in the further analysis and propensity score matching for study cohorts.

Demographics of patients included age, sex, body mass index (BMI), American Society of Anesthesiologists (ASA) grade, smoking status, alcohol consumption, and numbers of preoperative comorbidities. Additionally, intraoperative profiles included emergency surgery, types of surgery, anesthesia management, duration of surgery and anesthesia, blood loss, urine output, infusion of crystalloid or colloid fluid, blood transfusion, duration of systolic blood pressure > 140 mmHg, and duration of mean arterial pressure < 60 mmHg. Several laboratory tests included in the study were levels of hemoglobin, white blood cell (WBC) count, glucose (Glu), serum albumin, serum creatinine (Cre), blood potassium, blood sodium, alanine aminotransferase, and aspartate aminotransferase. Besides, preoperative medication of anticholinergic drugs, benzodiazepines, opioids; and intraoperative medication of glucocorticoid, dexmedetomidine, droperidol were recorded.

### Definitions of postoperative delirium

The diagnosis of postoperative delirium was first executed by capturing descriptive words in the electronic medical records. The inclusion criteria were the postoperative medical records of “mental status change”, “confusion”, “disorientation”, “agitation”, “delirium”, “inappropriate behavior”, “inattention”, “hallucinations”, “combative behavior”, “drowsy”, “slept poorly” and other similar meaning words in Chinese; and the postoperative medication of “quetiapine”, “olanzapine”, “haloperidol”, or “risperidone”. Exclusion criteria were preoperative recording of aforementioned symptoms or medications. Patients preliminarily diagnosed with POD were then manually confirmed by neurologists using the Diagnostic and Statistical Manual of Mental Disorders, fourth edition (DSM-IV) criteria.

### Statistical analysis

Continuous variables are expressed as mean ± standard deviation, and classified variables are expressed as count plus proportion. Categorical variables were analyzed by the chi-square test or Fisher exact test, and continuous variables were analyzed by the t-test or Mann-Whitney U nonparametric test. P-values below 0.05 were considered statistically significant. Multiple comparisons were adjusted by Bonferroni correction in post hoc analyses.

We performed propensity score matching (PSM) to avoid the influence of confounding factors on outcome events. We used a logistic regression model to estimate the propensity score to control known confounding variables; we set 0.2 as the caliper value. After propensity score matching, using delirium as the outcome, the univariate logistic regression model was used to determine the risk factors of 30 different comorbidities. Finally, eight comorbidities were selected for double or triple combinations, and the probability of the comorbidities in patients in the delirium and non-delirium groups was calculated, which can explain which several comorbidity combinations are prone to delirium. The likelihood of delirium in each comorbidity combination was calculated by combining the eight comorbidities in pairs. In addition, taking a single complication as a reference, the odd ratio (OR) value and p-value after its combination with other comorbidities were calculated by logistic regression to prove that the occurrence of two comorbidities was more prone to delirium than a single comorbidity. The R version 4.3.3 software was used for the corresponding statistical analysis.

## Results

### Patients characteristics

The medical records of 31,335 patients aged older than 65 years who underwent non-cardiac and non-neurological surgery from January 2014 to August 2019 at the First Medical Center of Chinese PLA General Hospital were retrospectively analyzed. Among the entire included population, 1034 developed postoperative delirium (POD) within seven days after surgery, and the average age of those diagnosed with POD was 74 years old; those without POD were 71 years old (data not shown). A cohort of 3102 patients without POD were matched by propensity score matching analysis at a ratio of 1:3 for those with POD. Except for ASA grades and numbers of preoperative comorbidities, perioperative demographics of those with and without POD were comparable after matching (Table 1).

**Table 1:** Demographics after propensity score matching

Variables	All patients (*n* = 4136)	Patients with POD (*n* = 1034)	Patients without POD (*n* = 3102)	*p* values
Age (Mean ± SD)	74	74.0 ± 6.6	74.3 ± 6.5	0.285
Sex (%)				0.744
Male	57.25%	587 (56.8%)	1781 (57.4%)	
Female	42.75%	447 (43.2%)	1321 (42.6%)	
BMI (Mean ± SD)	23.9	23.8 ± 3.9	23.8 ± 3.6	0.772
ASA (%)				< 0.001
I	0.68%	8 (0.8%)	20 (0.6%)	
II	63.49%	566 (54.7%)	2060 (66.4%)	
III	31.58%	378 (36.6%)	928 (29.9%)	
IV	3.05%	60 (5.8%)	66 (2.1%)	
V	1.21%	22 (2.1%)	28 (0.9%)	
Smoking Status (%)	26.64%	269 (26%)	833 (26.9%)	0.626
Alcohol Consumption (%)	22.92%	232 (22.4%)	716 (23.1%)	0.701
Number of Comorbidities				< 0.001
None	11.58%	88 (8.5%)	391 (12.6%)	
1	34.53%	334 (32.3%)	1094 (35.3%)	
2	28.22%	283 (27.4%)	884 (28.5%)	
≥ 3	25.68%	329 (31.8%)	733 (23.6%)	
Emergency surgery (%)	12.16%	143 (13.8%)	360 (11.6%)	0.066
Type of surgery				
E.N.T (%)	2.18%	24 (2.3%)	66 (2.1%)	0.806
Gynecology (%)	2.42%	24 (2.3%)	76 (2.5%)	0.907
Hepatopancreatobiliary and gastrointestinal surgery (%)	41.08%	417 (40.3%)	1282 (41.3%)	0.597
Orthopedic surgery (%)	27.39%	283 (27.4%)	850 (27.4%)	1.000
Thyroid and Brest (%)	1.31%	14 (1.4%)	40 (1.3%)	1.000
Stomatology (%)	2.56%	29 (2.8%)	77 (2.5%)	0.649
Urinary surgery (%)	6.75%	64 (6.2%)	215 (6.9%)	0.452
Thoracic surgery (%)	4.74%	49 (4.7%)	147 (4.7%)	1.000
Vascular surgery (%)	4.79%	58 (5.6%)	140 (4.5%)	0.178
Plastic surgery(%)	0.15%	1 (0.1%)	5 (0.2%)	1.000
Other (%)	6.65%	71 (6.9%)	204 (6.6%)	0.801
Type of anesthesia				
Monitored anesthesia care (%)	1.76%	19 (1.8%)	54 (1.7%)	0.946
General anesthesia combined with other anesthesia (%)	11.19%	112 (10.8%)	351 (11.3%)	0.711
General anesthesia (%)	83.97%	870 (84.1%)	2603 (83.9%)	0.903
Nerve block (%)	2.66%	26 (2.5%)	84 (2.7%)	0.823
Spinal or epidural anesthesia (%)	0.41%	7 (0.7%)	10 (0.3%)	0.207
Duration of surgery (Mean ± SD)	187	187.0 ± 107.2	186.9 ± 115.6	0.980
Duration of anesthesia (Mean ± SD)	236	238.2 ± 113.4	235.0 ± 121.9	0.446
Blood loss (Mean ± SD)	293	319.4 ± 707.0	284.5 ± 646.4	0.161
Urine (Mean ± SD)	463	468.4 ± 470.9	461.4 ± 474.0	0.680
Crystalloid (Mean ± SD)	1771	1785.9 ± 894.7	1766.0 ± 934.5	0.548
Colloid (Mean ± SD)	573	589.3 ± 531.5	568.2 ± 557.4	0.287
Blood plasma (Mean ± SD)	58	66.3 ± 174.7	55.2 ± 207.1	0.093
Blood transfusion (%)	13.10%	139 (13.4%)	403 (13%)	0.750
Duration of SBP > 140 mmHg (Mean ± SD)	21.5	20.8 ± 31.7	21.7 ± 34.8	0.470
Duration of MAP < 60 mmHg (Mean ± SD)	7.2	7.1 ± 13.6	7.2 ± 12.3	0.845

Since inflammation was considered a significant risk factor for POD, preoperative laboratory testing was also taken into PSM analysis, and variables, including inflammation-related white blood cell counts, showed no differences after matching. In addition, preoperative usage of anticholinergic drugs, benzodiazepines, opioids, and intraoperative usage of corticosteroids, dexamethasone, and haloperidol, which might affect the incidence of POD, were invariant between cohorts with and without POD (Table 2).

**Table 2:** Laboratory analysis and preoperative medications after propensity score matching

Variables	All patients (*n* = 4136)	Patients with POD (*n* = 1034)	Patients without POD (*n* = 3102)	*p* values
Hemoglobin (Mean ± SD)	121.5	121.4 ± 20.6	121.5 ± 19.6	0.948
WBC count (%)				0.230
no more than 4	6.14%	71 (6.9%)	183 (5.9%)	
more than 4, no more than 10	79.30%	801 (77.5%)	2479 (79.9%)	
more than 10	14.56%	162 (15.7%)	440 (14.2%)	
Glucose (Mean ± SD)	6.2	6.3 ± 2.5	6.2 ± 2.5	0.141
Serum albumin (Mean ± SD)	37.4	37.4 ± 5.0	37.5 ± 4.7	0.711
Cre (Mean ± SD)	80.7	80.6 ± 48.6	80.8 ± 57.1	0.942
Blood potassium (Mean ± SD)	4.1	4.1 ± 0.4	4.1 ± 0.4	0.813
Blood sodium (Mean ± SD)	140	139.9 ± 4.4	140.0 ± 4.0	0.414
AST (Mean ± SD)	25.4	25.6 ± 34.5	25.4 ± 39.1	0.888
ALT (Mean ± SD)	23.7	23.8 ± 35.7	23.7 ± 31.9	0.949
Preoperative medication				
Anticholinergic agents (%)	53.19%	545 (52.7%)	1655 (53.4%)	0.746
Benzodiazepines (%)	23.57%	243 (23.5%)	732 (23.6%)	0.983
Opioids (%)	7.83%	80 (7.7%)	244 (7.9%)	0.947
Intraoperative medication				
Glucocorticoid (%)	62.55%	645 (62.4%)	1942 (62.6%)	0.926
Dexmedetomidine (Mean ± SD)	0.93	1.0 ± 3.5	0.9 ± 5.0	0.750
Droperidol (Mean ± SD)	0.1	0.1 ± 0.3	0.1 ± 0.3	0.326

### Identification of univariate preoperative comorbidities as risk factors for POD

Univariate risk factors were selected by logistic regression, and eight preoperative comorbidities, including hypertension (OR 1.22 [1.06–1.41], *p* = 0.005), diabetes (OR 1.18 [1.00-1.38], *p* = 0.043), atrial fibrillation (OR 1.92 [1.34–2.75], *p* < 0.001), cerebrovascular disease (OR 1.59 [1.30–1.93], *p* < 0.001), Parkinson’s disease (OR 2.30 [1.26–4.20], *P* = 0.006), epilepsy (OR 4.32 [1.64–11.37], *p* = 0.006), depression (OR 3.03 [1.48–6.22], *p* = 0.003), and chronic obstructive pulmonary disease (COPD) (OR 2.00 [1.54–2.61], *p* < 0.001) were more common in patients diagnosed with POD after surgery than those without POD (Table 3).

**Table 3:** Univariate analysis of preoperative comorbidities

Comorbidity	All patients	Patients with POD (*n* = 1034)	Patients without POD (*n* = 3102)	OR (95% CI, *p*)
Hypertension	48.53%	541 (52.3%)	1466 (47.3%)	1.22 (1.06–1.41, *p* = 0.005)
Diabetes	26.14%	295 (28.5%)	786 (25.3%)	1.18 (1.00-1.38, *p* = 0.043)
Atrial fibrillation	3.14%	50 (4.8%)	80 (2.6%)	1.92 (1.34–2.75, *p* < 0.001)
Cerebrovascular disease	12.77%	175 (16.9%)	353 (11.4%)	1.59 (1.30–1.93, *p* < 0.001)
Parkinson’s disease	1.06%	19 (1.8%)	25 (0.8%)	2.30 (1.26–4.20, *p* = 0.006)
Epilepsy	0.41%	10 (1%)	7 (0.2%)	4.32 (1.64–11.37, *p* = 0.003)
Depression	0.73%	15 (1.5%)	15 (0.5%)	3.03 (1.48–6.22, *p* = 0.003)
COPD	6.09%	98 (9.5%)	154 (5%)	2.00 (1.54–2.61, *p* < 0.001)

### Identification of combinations of preoperative comorbidities as risk factors for POD

**Fig. 1:**
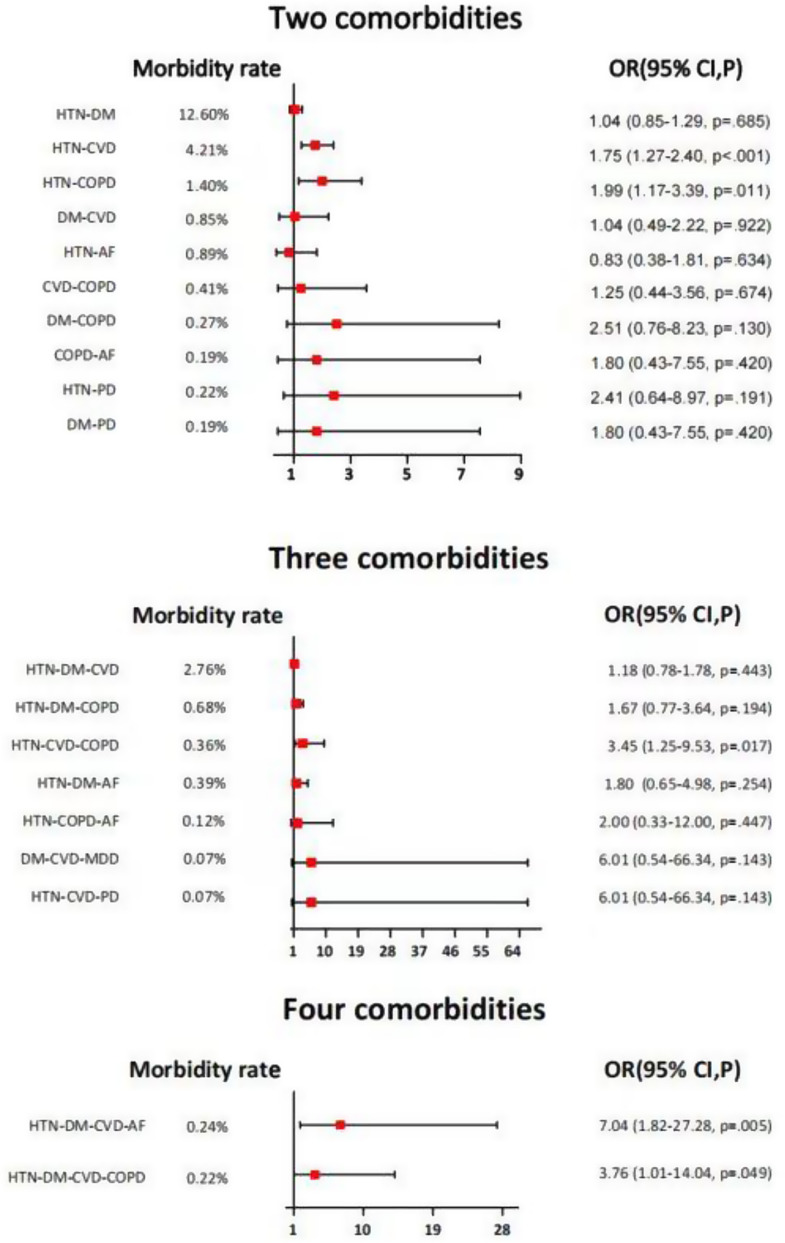
Comparisons of the combinations of preoperative comorbidities

Since older patients accepted surgeries might have more than one preoperative comorbidity, we then analyze the incidence of different combinations of comorbidities in cohorts with and without POD. Overall, 28.22% of all included older patients suffered from two comorbidities, and 25.68% suffered from three or more comorbidities (Table 1). Patients with POD were more likely to concurrently suffer from the combinations of hypertension and cerebrovascular disease (OR 1.75 [1.27–2.40], *p* < 0.001), the combinations of hypertension and COPD (OR 1.99 [1.17–3.39], *p* = 0.011), the combinations of hypertension, cerebrovascular disease and COPD (OR 3.45 [1.25–9.53], *p* = 0.017), the combinations of hypertension, diabetes, cerebrovascular disease, and atrial fibrillation (OR 7.04 [1.82–27.28], *p* = 0.005), the combinations of hypertension, diabetes, cerebrovascular disease, and COPD (OR 3.76 [1.01–14.04], *p* = 0.049) (Fig. 1).

### Associations between combinations of two preoperative comorbidities and POD

**Table 4:**
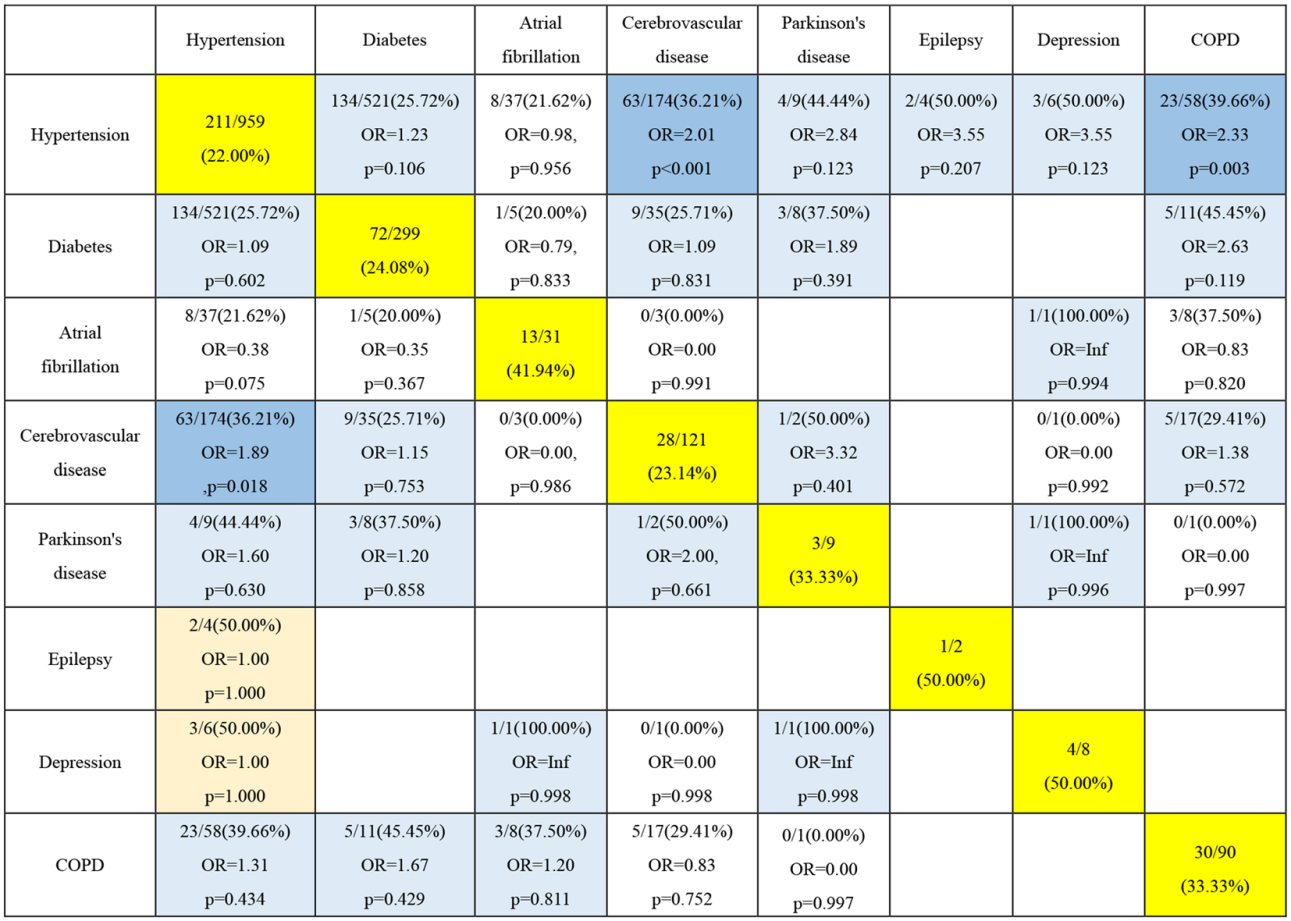
Incidence of postoperative delirium in the population with two different preoperative comorbidities

Patients with both cerebrovascular disease and hypertension were more likely to experience delirium and have statistical differences compared to patients with cerebrovascular disease and hypertension alone. Patients with both hypertension and COPD were more likely to experience delirium and have statistical differences compared to patients with only hypertension. From the table as a whole, it can be discerned that patients with two comorbidities were more prone to delirium than patients with one comorbidity. Although there was no statistical difference, the incidence of patients with two comorbidities was higher than that of patients with only one comorbidity, and the OR value was more significant than 1. This is consistent with clinical significance (Table 4).

## Discussion

There are currently few effective treatments for POD except for minimizing its risk factors, and older patients with preoperative comorbidities are at higher risk for developing POD [1, 2]. Since the older patients were more likely to have multiple comorbidities, identifying the specific comorbidities and their combinations, which were associated with POD, would help optimize the preoperative health status to reduce the risk of POD [4]. The current retrospective matched cohort study identified several preoperative comorbidities that could increase the risk of POD and further found that the older patients comorbid with combinations of hypertension and cerebrovascular disease, or hypertension and COPD, might be more susceptible to developing POD after surgery [5, 8].

Consistent with previous studies, we found that higher ASA health status classes, which indicated more preoperative comorbidities, were associated with a higher risk of POD [3, 11]. As for univariate comorbidity, we found it would be easier to experience POD for older patients with preoperative hypertension, diabetes, atrial fibrillation, cerebrovascular disease, Parkinson’s disease, epilepsy, depression, or COPD [2, 5].

It has been reported that a higher comorbidity burden, especially the psychiatric diseases, would increase the risk of POD [1]. Similarly, we identified cerebrovascular diseases, Parkinson’s disease, epilepsy, and depression as risk factors for POD. These diseases might lead to a central neural system malfunction preoperatively, making the patients’ brains more vulnerable to general anesthesia and surgery stress [4]. Besides, the pathophysiological changes of POD are focused on the involvement of neuroinflammation, neurovascular, neurotransmitter, or neurometabolic dysfunction [8]. Parkinson’s disease associated with dopaminergic system impairment^12^], epilepsy with glutamatergic system hyperexcitability [13], and depression with monoaminergic system disturbance [14], might magnify the influence of anesthetics on the neurotransmitter system to cause POD [3]. Older patients with hypertension, diabetes, or atrial fibrillation were often at a potential disadvantage from systematic vascular damage, and the cerebrovascular fragility would contribute to postoperative psychiatric disorders like delirium or cognitive decline [11, 15].

Chronic anoxic status caused by COPD puts the central nervous system of older patients in danger of hypoxia and impedes their resilience to surgical or anesthetic stress [16, 17]. Indeed, we found that COPD was associated with the occurrence of POD. It has been suggested that preoperative COPD increases the probability of postoperative respiratory complications, including difficult extubation or respiratory system infections, which could lead to delirium [18]. Moreover, smoking, as a primary cause of COPD, was also reported to display a positive correlation with POD [19, 20]. Together with the previous findings, the identification of COPD as a significant risk factor in our study may further strengthen the oxygenation throughout the whole perioperative management for the prevention of POD.

Importantly, since older surgical patients were often affected by more than one comorbidity, we further recognized that several preoperative comorbidities were associated with POD combinations. Hypertension was the only comorbidity as a component of all the combinations related to POD, suggesting its dominant role in the susceptibility of POD [21, 22]. Although the eight preoperative comorbidities all displayed a correlation with POD in the univariate analysis, only the combinations of hypertension and cerebral vascular disease, or hypertension and COPD, were significantly associated with POD. It was noticeable that with the highest morbidity rate, older patients suffering from the combination of hypertension and diabetes were not prone to POD. The reason for this discrepancy could be that hypertension or diabetes has become relatively controllable diseases [23, 24], and well-controlled hypertension and diabetes without substantive functional limitations before surgery had a minor impact on POD. The relatively low morbidity rate of other combinations of two preoperative comorbidities might be attributed to their insignificant correlation with POD.

The current study has several limitations. The sample size would be small for combinations of three or more preoperative comorbidities. However, with the aging population, older patients would be more likely to suffer from more preoperative comorbidities [6, 7]. Therefore, the combinations of more than three comorbidities that could lead to POD still need to be investigated in future studies. Due to the popularization of long-term health management for older patients, most surgical patients have been under treatment for their preoperative comorbidities, and these comorbidities may be well controlled [25]. Although for patients with hypertension, diabetes or other comorbidities undertaking elective surgeries, preoperative conditions were required to be controlled in a relatively stable status, we did not evaluate the treatment conditions of preoperative comorbidities, which might be an essential factor to study in the future. Besides, the current retrospective study screened patients with POD from electronic medical records, which may weaken the diagnostic reliability. Prospective study design with verified delirium screening tools like Confusion Assessment Methods (CAM) are still needed in further study for more consolidated conclusions.

## Conclusion

There are currently few effective treatments for POD except for minimizing its risk factors. This retrospective propensity-matched cohort study found that preoperative hypertension, diabetes, atrial fibrillation, cerebrovascular disease, Parkinson’s disease, epilepsy, depression, or COPD are associated with a higher risk of POD in older surgical patients. Furthermore, older patients suffering from several combinations of preoperative comorbidities were more likely to develop POD, and hypertension plays a central role in these combinations. Our results further emphasized the importance of the management of preoperative comorbidities in older surgical patients. And we put forward several preoperative comorbidities and their combinations to be prioritized throughout the perioperative management.

## Data Availability

All data presented in this study are available on reasonable request from the corresponding author.

## Abbreviations

ASA: American Society of Anesthesiologists
BMI: Body mass index
CCI: Charlson Comorbidity Index
COPD: Chronic obstructive pulmonary disease
Cre: Serum creatinine
DSM-IV: Diagnostic and Statistical Manual of Mental Disorders, fourth edition
Glu: Glucose
OR: Odd ratio
POD: Postoperative delirium
PSM: Propensity score matching
WBC: White blood cell
